# Data on the standardization of a cyclohexanone-responsive expression system for Gram-negative bacteria

**DOI:** 10.1016/j.dib.2016.01.022

**Published:** 2016-01-22

**Authors:** Ilaria Benedetti, Pablo I. Nikel, Víctor de Lorenzo

**Affiliations:** Systems and Synthetic Biology Program, Centro Nacional de Biotecnología (CNB-CSIC), Madrid 28049, Spain

**Keywords:** Expression system, Heterologous gene expression, Metabolic engineering, Synthetic biology, *Escherichia coli*, *Pseudomonas putida*

## Abstract

Engineering of robust microbial cell factories requires the use of dedicated genetic tools somewhat different from those traditionally used for laboratory-adapted microorganisms. We have edited and formatted the ChnR/*P*_*chnB*_ regulatory node of *Acinetobacter johnsonii* to ease the targeted engineering of ectopic gene expression in Gram-negative bacteria. The proposed compositional standard was thoroughly verified with a monomeric and superfolder green fluorescent protein (msf•GFP) in *Escherichia coli*. The expression data presented reflect a tightly controlled transcription initiation signal in response to cyclohexanone. Data in this article are related to the research paper “*Genetic programming of catalytic* Pseudomonas putida *biofilms for boosting biodegradation of haloalkanes*” [1].

**SPECIFICATIONS TABLE**

**Table:** 

Subject area	*Biology*
More specific subject area	*Metabolic Engineering*
Type of data	*Figures and Table*
How data was acquired	*Flow cytometry*
Data format	*Analyzed*
Experimental factors	*Assembly and validation of a standard expression vector for Gram-negative bacteria using regulatory elements from Acinetobacter johnsonii*
Experimental features	*Molecular Biology and Synthetic Biology methodologies (DNA synthesis, PCR, enzyme restriction, DNA ligation), flow cytometry*
Data source location	*Madrid, Spain*
Data accessibility	*Data is with this article*

**VALUE OF THE DATA**

• Standardized vector designed for tightly regulated gene expression in Gram-negative bacteria.
• Regulatory elements from *Acinetobacter johnsonii* (ChnR transcriptional regulator and *P*_*chnB*_ promoter) edited, formatted, and assembled in a minimal DNA segment adopting a Synthetic Biology standard.
• Responsiveness of the expression system to the inducer cyclohexanone demonstrated by using GFP as a reporter.
• The DNA standard described in this dataset could be used as a benchmark for future research on gene expression in Gram-negative bacteria.

## 1 Data

**Fig. 1:**
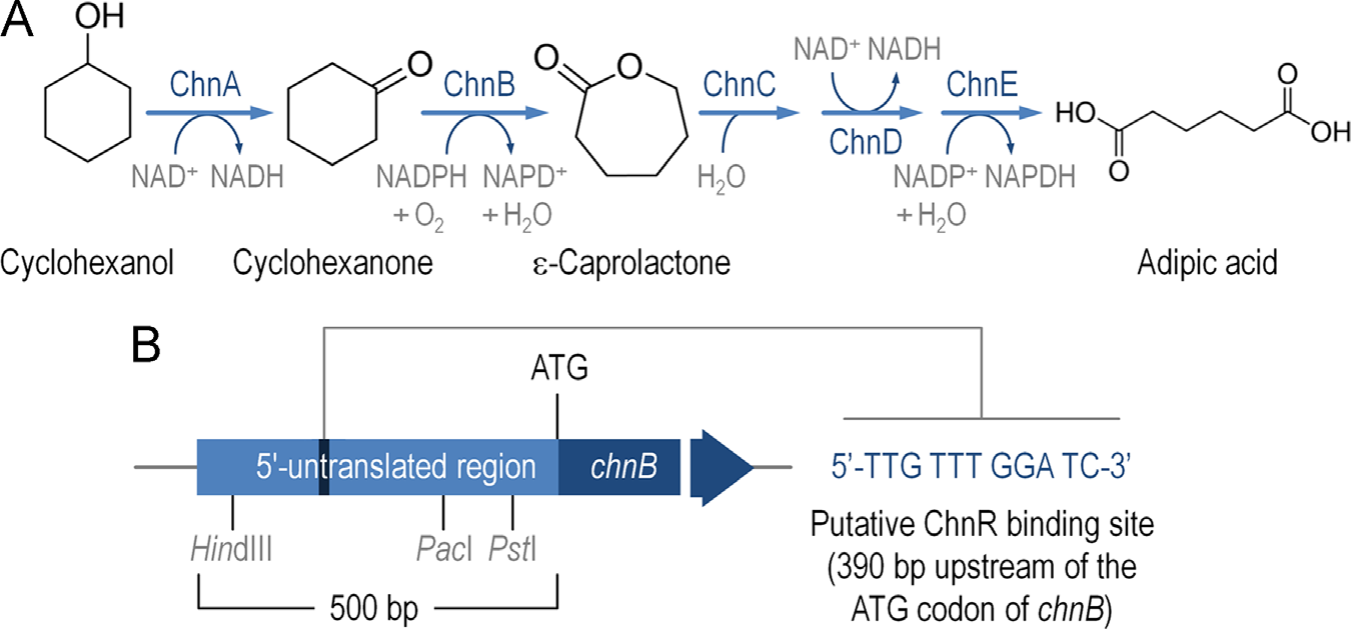
Design and construction of a standardized vector carrying the ChnR/*P*_*chnB*_ expression system. (A) Proposed biochemical pathway for the conversion of cyclohexanol into adipic acid by *Acinetobacter johnsonii* NCIMB 9871 [18], source of the genetic elements for the expression system described in this dataset. Enzymes involved in the biodegradation pathway have been identified by Cheng et al. [10] as ChnA, alcohol dehydrogenase; ChnB, cyclohexanone monooxygenase; ChnC, hydrolase; ChnD, alcohol dehydrogenase; and ChnE, aldehyde dehydrogenase. The cofactor specificity of the enzymes is indicated in the pathway. (B) DNA fragment used for the construction of the expression system described in this dataset. The 500-bp DNA fragment preceding the ATG codon of the *chnB* gene was edited in order to eliminate the three restriction targets also present in all pSEVA vectors [2], [17]. The putative ChnR binding site [12] is indicated.

**Fig. 2:**
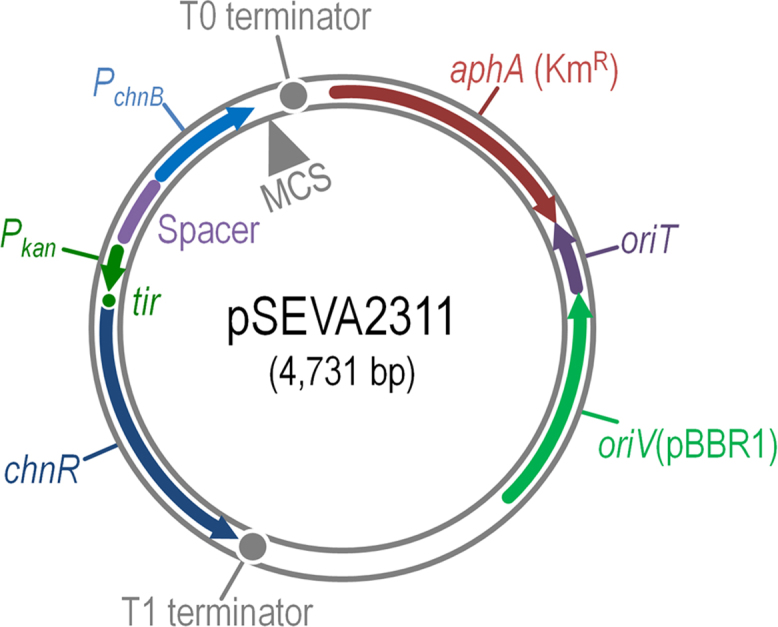
Physical map of plasmid pSEVA2311, carrying the functional elements of the ChnR/*P*_*chnB*_ expression system. The main functional elements of the plasmid include [i] the gene encoding the ChnR positive regulator from *Acinetobacter johnsonii* NCIMB 9871, [ii] the strong *P*_*kan*_ promoter, driving the constitutive expression of *chnR*, [iii] a spacer sequence to insulate the expression of adjacent DNA sequences, [iv] a *tir* motif, and [v] the *P*_*chnB*_ promoter, that is activated by ChnR. The T0 and T1 strong terminators insulate the transcription of the functional DNA segment carrying the expression system. MCS, multiple cloning site; Km^R^, kanamycin-resistance determinant.

**Fig. 3:**
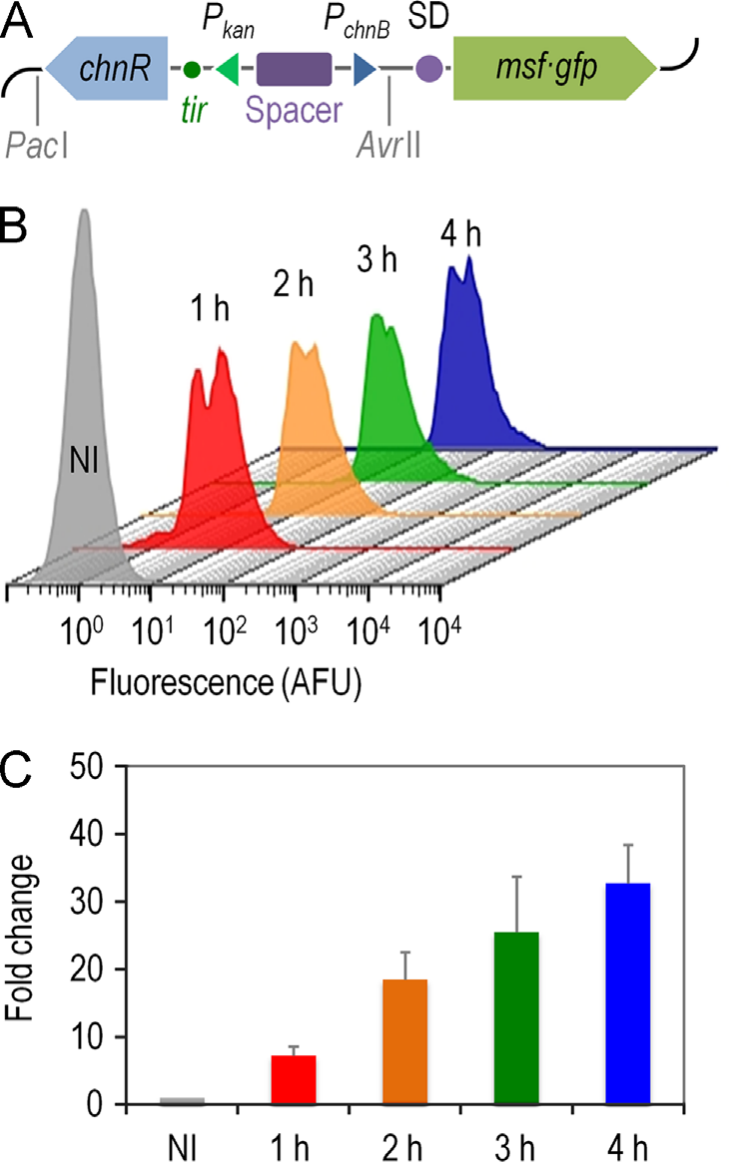
Experimental validation of the pSCM expression plasmid. (A) Functional elements of the plasmid include [i] the gene encoding the ChnR positive regulator from *Acinetobacter johnsonii* NCIMB 9871, [ii] the strong *P*_*kan*_ promoter, driving the expression of *chnR*, [iii] a spacer sequence to insulate the expression of adjacent DNA sequences, [iv] a *tir* motif, and [v] the *P*_*chnB*_ promoter, that is activated by ChnR. Note that the ChnR/*P*_*chnB*_ expression system can be inserted in any other SEVA plasmid as a *Pac*I/*Avr*II segment. The gene encoding the monomeric and superfolder green fluorescent protein (msf•GFP), preceded by a synthetic Shine–Dalgarno (SD) motif, was cloned into plasmid pSEVA2311 (Table 1) originating plasmid pSCM, used for validation purposes. Elements in this outline are not drawn to scale. (B) Flow cytometry assessment of msf•GFP fluorescence stemming from the ChnR/*P*_*chnB*_ expression system. *Escherichia coli* BW25113/pSCM was grown until mid-exponential phase in semi-synthetic M9CAG medium, and at this point cyclohexanone was added at 1 mM to the cultures. Samples were withdrawn and analyzed by flow cytometry in 1 h intervals thereafter. AFU, arbitrary fluorescence units; NI, non-induced control. (C) Induction profile of *E. coli* BW25113/pSCM grown in semi-synthetic M9CAG medium in response to 1 mM cyclohexanone. The output of the *P*_*chnB*_ promoter was calculated by normalizing the average fluorescence levels (geometric *x*-mean) of induced populations by the fluorescence levels of the control samples. At least 20,000 cells were analyzed in each assay. Bars represent the mean values of the corresponding parameter±standard deviation of triplicate measurements from at least four independent experiments. NI, non-induced control.

A cyclohexanone-responsive expression platform was designed based on elements of the cyclohexanol biodegradation pathway of *Acinetobacter johnsonii* (Fig. 1A). The segments bearing the complete *chnR* and *P*_*chnB*_ promoter DNA of *A. johnsonii* NCIMB 9871 were edited *in silico* to obtain a standardized SEVA (*S*tandard *E*uropean *V*ector *A*rchitecture [2]) expression cargo (Fig. 1B), and assembled to yield plasmid pSEVA2311 (Fig. 2 and Table 1). Expression data were generated to validate this plasmid. The gene encoding the monomeric and superfolder green fluorescent protein (msf•GFP) was inserted in vector pSEVA2311 (Fig. 3A), and the transcriptional activation of the ChnR/*P*_*chnB*_ expression system upon addition of cyclohexanone was evaluated in a wild-type *Escherichia coli* strain (Fig. 3B and C). Key features of this expression vector include [i] a very low expression level in the absence of inducer, [ii] high transcriptional capacity, [iii] an induction kinetics very similar in both minimal and rich culture media, and [iv] linear accumulation of the reporter product along time.

## 2 Experimental design, materials and methods

### 2.1 Bacterial strains and culture conditions

Bacterial strains used in this study are listed in Table 1. *E. coli* CC118 was used as the host for plasmid constructs and it was routinely grown at 37 °C in LB medium [3]. For single-cell fluorescence determination by flow cytometry, cells were grown in the semi-synthetic M9CAG medium, which contains the same salts as for M9 minimal medium [3], 0.1% (w/v) acid casein hydrolysate (Becton-Dickinson Diagnostics Co., Sparks, MD, USA), 2 mM MgSO_4_, 0.1 mM CaCl_2_, 0.05% (w/v) vitamin B1, and 0.4% (w/v) glucose as the sole carbon and energy source. Kanamycin (Km, 50 μg ml^−1^) was added to the culture media whenever required. Growth was estimated by measuring the optical density at 600 nm (OD_600_) after diluting the culture whenever needed [4], [5], [6]. Shaken-flask cultures were set in 125-ml Erlenmeyer flasks containing culture medium up to one-fifth of their nominal volume and continuously agitated at 170 rpm. Cyclohexanone was directly added to the cultures as an inducer of the ChnR/*P*_*chnB*_ system at 1 mM.

### 2.2 DNA techniques, plasmid design and construction, and validation of a standardized expression system with regulatory parts from Acinetobacter johnsonii NCIMB 9871

All the plasmids used in this study are listed in Table 1. DNA amplification by PCR, digestion with restriction enzymes, ligation, and other standard cloning procedures followed well established protocols [3], [7], [8], [9] and specific instructions from the manufacturers. All plasmid constructs were confirmed by Sanger DNA sequencing (Secugen SL, Madrid, Spain).

To obtain vector pSEVA2311, an expression plasmid containing the *P*_*chnB*_ promoter and the gene encoding the cyclohexanone-responsive ChnR transcriptional regulator, a DNA fragment carrying both *chnR* and *P*_*chnB*_ was designed as a SEVA expression cargo (i.e., *Pac*I/*Avr*II), and synthesized *de novo* by GeneCust Europe S.A. (Dudelange, Luxembourg). The DNA sequence encoding ChnR, including a putative binding site for ChnR upstream to the *P*_*chnB*_ promoter, was taken from the genome of *A. johnsonii* NCIMB 9871 [10], [11], [12]. The recognition site for *Hin*dIII, *Pac*I, and *Pst*I in this synthetic DNA fragment were manually edited to erase them, as these restriction targets are present in the multiple cloning site of all pSEVA plasmids (Fig. 1B). A DNA fragment was amplified using oligonucleotides *chnR*-F (5′-TTT TTT AAT TAA TCA AAA AAC AAT AGA GGA GAC TGA ATT TTC-3′, recognition site for *Pac*I underlined) and *chnR*-R (5′-TTT TGC TAG CAT GAG CAC AGA CAA AGC AAA TAC-3′, recognition site for *Nhe*I underlined) from the synthetic DNA fragment described above, and sub-cloned into vector pSEVA231 as a *Pac*I/*Nhe*I fragment. The constitutive expression of *chnR* was achieved by inserting a *Nhe*I/*Avr*II fragment that spans a 150-bp long linker sequence and the strong, constitutive *P*_*kan*_ promoter [13] along with the *tir* motif (5′-GAT TAA CTT TAT AAG GAG GAA AAA-3′ [14]), giving rise to the intermediate plasmid pSEVA231-ChnR (Table 1). This vector was inserted with the *P*_*chnB*_ promoter, originating pSEVA2311 (Fig. 2 and Table 1). Another version of the expression vector was also constructed, but using a plasmid backbone bearing the pRO1600/ColE1 origin of replication, giving rise to pSEVA2411 (Table 1). For the experimental validation of the ChnR/*P*_*chnB*_ expression system, the gene encoding the monomeric and superfolder green fluorescent protein (msf•GFP) was excised from plasmid pSEVA237M as a *Hin*dIII/*Spe*I fragment and cloned into pSEVA2311, giving rise to plasmid pSCM (Table 1 and Fig. 3).

### 2.3 Single-cell analysis by flow cytometry

A MACSQuant^TM^ VYB cytometer (Miltenyi Biotec, Bergisch Gladbach, Germany) was used for msf•GFP analysis and quantification as indicated elsewhere [15]. An Ar laser, diode-pumped solid state, was used to excite msf•GFP at 488 nm and the fluorescence signal was recovered with a 525±40 nm band-pass filter. Plasmid pSCM was introduced in wild-type *Escherichia coli* BW25113 by chemical transformation [3]. Recombinant cells were grown overnight in semi-synthetic M9CAG medium with the appropriate carbon source and antibiotics, diluted 1/100 in fresh M9CAG culture medium (initial OD_600_ of ca. 0.05), and further incubated until the cultures reached the mid-exponential phase (OD_600_=0.5). At this point, cells were divided into two samples; one of them was induced by addition of cyclohexanone at 1 mM, and the other was kept as a non-induced control. Cultures were further incubated as described above and an aliquot of each sample was withdrawn each hour after the induction point, and stored on ice until analysis. The flow cytometry analysis was executed on at least 20,000 cells and the data was processed using FlowJo v. 9.6.2 software (FlowJo LLC, Ashland, OR, USA) [16]. Data regarding expression levels and induction kinetics for *E. coli* strains carrying plasmid pSCM were similar in LB medium and M9CAG medium (data not shown).

### 2.4 Chemicals and reagents

Unless stated otherwise, all chemicals and inducers were purchased from Sigma-Aldrich Co. (St. Louis, MO), while flow cytometry materials (buffers and calibration beads) were purchased from Miltenyi Biotec GmbH.

## 3 Material availability and repository

The expression vectors described in this work are part of the SEVA initiative (http://wwwuser.cnb.csic.es/~seva/, [2], [17]) and are available free of charge upon request.

## Figures and Tables

**Table 1:** Bacterial strains and plasmids used in this study.

Bacterial strain	Relevant characteristics⁎	Reference
*Escherichia coli*		
CC118	Cloning host; Δ(*ara*-*leu*) *araD* Δ*lacX174 galE galK phoA thiE1 rpsE rpoB*(Rif ^R^) *argE*(Am) *recA1*	[19]
BW25113	Wild-type strain; F^–^ λ^–^ Δ(*araD-araB*)*567* Δ*lacZ4787*(::*rrnB-3*) *rph-1* Δ(*rhaD-rhaB*)*568 hsdR514*	[20]
Plasmids		
pSEVA231	Km^R^; *oriV*(pBBR1), standard multiple cloning site	[2]
pSEVA231-ChnR	Km^R^; pSEVA231 carrying *chnR* (encodes the ChnR transcriptional regulator from *Acinetobacter johnsonii*)	This study
pSEVA237M	Km^R^; *oriV*(pBBR1), promoterless *msf•GFP*	This study
pSEVA2311	Km^R^; *oriV*(pBBR1), *chnR*, *P*_*chnB*_; standardized, cyclohexanone-responsive expression vector	This study
pSEVA2411	Km^R^; *oriV*(pRO1600/ColE1), *chnR*, *P*_*chnB*_; standardized, cyclohexanone-responsive expression vector	This study
pSCM	Km^R^; *oriV*(pBBR1), *chnR*, *P*_*chnB*_→*msf•GFP*	This study

⁎ The abbreviations used in this table are as follows: Km, kanamycin; Sm, streptomycin; Rif, rifampicin; *msf*•*gfp*, gene encoding the monomeric and superfolder green fluorescent protein (msf*•*GFP).
